# Bridging Connection & Care: AI-Modified Activities for Residents

**DOI:** 10.1093/geroni/igaf122.2242

**Published:** 2025-12-31

**Authors:** ZaKiyah Timmons-Crear, Melinda Heinz

**Affiliations:** University of Northern Iowa, waterloo, Iowa, United States; University of Northern Iowa, Cedar Falls, Iowa, United States

## Abstract

This study explored the effectiveness of using artificial intelligence (AI) to modify activities in care facilities based on vision, hearing, mobility impairments, and for those living with dementia. AI has become a widespread tool, although its applications in care facilities remains unexplored. Two programs (e.g., Co-Pilot and ChatGPT) were used to explore their effectiveness in modifying activities from four different care facilities (e.g., independent living, assisted living, nursing home, and memory care). Actual activity calendars from each facility were used. In the first iteration, our prompt was “In a [type of care facility] how would you modify [type of activity] for someone who has [dementia, mobility, hearing, or vision impairment]. We noted that ChatGPT provided significantly more detail about how the activity could be modified and why that would be important (e.g., explaining why using bright colors and textures might help to provide increased sensory stimulation for those living with dementia). In a second iteration, we asked Co-Pilot and ChatGPT to modify the activities if the facility was short-staffed. Both AI platforms suggested allowing residents more independence with the activities. However, for some areas, such as memory care units, it may not be feasible for residents to complete activities independently due to their cognitive functioning. These findings suggest that AI may be most useful for providing employees guidance with tailoring activities to individual needs. However, AI lacked critical thinking when suggesting that residents could complete activities with more independence if the facility were short staffed.

